# Magnetization reversal of Co/Pd multilayers on nanoporous templates

**DOI:** 10.1186/1556-276X-7-41

**Published:** 2012-01-05

**Authors:** Chien-Chih Huang, Chin-Chung Yu, Shih-Yuan Chen, Yeong-Der Yao, Jun-Yang Lai

**Affiliations:** 1 Department of Applied Physics, National University of Kaohsiung, Kaohsiung 811, Taiwan, Republic of China; 2 Department of Materials Science and Engineering, National Tsing-Hua University, Hsin-Chu 300, Taiwan, Republic of China; 3 Graduate Institute of Applied Science and Engineering, Fu Jen Catholic University, Taipei 24205, Taiwan, Republic of China; 4 Department of Applied Physics, National Ping Tung University of Education, Ping Tung 900, Taiwan, Republic of China

**Keywords:** Co/Pd, porous anodized aluminum oxide, magnetization reversal

## Abstract

By making use of an e-beam deposition system, the [Co(2 Å)/Pd(10 Å)]_15 _multilayers were prepared on a Si(100) substrate and anodized aluminum oxide [AAO] templates with average pore diameters of around 185, 95, and 40 nm. The mechanism of magnetization reversal of the Co/Pd multilayers was investigated. Wall motion was observed on the Co/Pd multilayers grown on the Si substrate. A combination of wall motion and domain rotation was found in the sample grown on the AAO template with a 185-nm pore diameter. For the samples grown on the AAO templates with pore diameters of around 95 and 40 nm, the reversal mechanism was dominated by domain rotation. The rotational reversal was mainly contributed from the underlying nanoporous AAO templates that provided an additional pinning effect.

**PACS: **75.30.Gw, magnetic anisotropy; 78.67.Rb, nanoporous materials; 75.60.Jk, magnetization reversal mechanisms.

## Introduction

In the last decade, patterned magnetic nanostructures have undergone a rapid development because of their potential applications in future high-density magnetic recording media [1-4]. Nowadays, the FePt film with L1_0 _phase has received significant attention owing to its excellent magnetic properties, presenting large values of the saturation magnetization (approximately 1,100 emu/cm^3^), the perpendicular magnetocrystalline anisotropy constant (approximately 10^8 ^erg/cm^3^), the coercivity, and the high environmental stability [5-7]. Another choice for the perpendicular recording is ferromagnetic/non-ferromagnetic multilayers. For example, Co/Pt and Co/Pd are also candidates for the perpendicular recording media of the next generation. As the areal density of today's hard disk drives keeps approaching to the limit of current technology, exploring new techniques to obtain higher and higher density is inevitable [8-10]. There have been significant research efforts focusing on the patterned magnetic recording medium with its density larger than 1 Tbit/in^2^. In addition, the percolated recording media has been investigated because of its high thermal stability and low-medium noise [11-13]. As a result, the magnetic nanomaterial fabricated through nanoporous anodized aluminum oxide [AAO] is a good candidate in the pursuit of patterned recording media.

In this article, we fabricated the Co/Pd multilayers on a Si(100) substrate and on nanoporous AAO templates with different pore diameters using an electron beam evaporator. We attempt to study the magnetization reversal and domain structures of the Co/Pd multilayers on nanoporous templates.

## Experimental details

Before film deposition, the Si(100) substrate was cleaned in acetone and alcohol solutions using an ultrasonic bath. Three types of nanoporous AAO templates were purchased from Whatman International Ltd (Maidstone, UK) and have different pore diameters: 185, 95, and 40 nm. Prior to the growth of Co/Pd multilayers, a 10-nm-thick Pd buffer layer was grown on the Si substrate and AAO templates. The [Co(2 Å)/Pd(10 Å)]_15 _multilayers were then deposited on the Pd buffer layer by an e-beam system with a growth pressure of around 5 × 10^-8 ^Torr. During the deposition, the temperature of the substrates and the deposition rate were kept at 150°C and 0.1 Å/s, respectively.

The surface morphology of AAO templates was examined using a scanning electron microscope [SEM] (JSM-6390, JEOL, Tokyo, Japan). The magnetic hysteresis loops were measured at room temperature using a vibrating sample magnetometer (7407, Lake Shore, Westerville, OH, USA) with an applied field up to 1 T. The domain structures were imaged using a magnetic force microscope [MFM] (XE-100, Park Systems, Suwon, South Korea) under a lift mode operation. The magnetic tip used for the MFM measurements was purchased from Nanosensors Inc. (Neuchatel, Switzerland) with a catalog number of ppp-MFMR. The MFM tip radius of curvature was less than 50 nm. It guaranteed a magnetic resolution better than 50 nm. Because the AAO templates were too thin (thickness of approximately 60 μm) for handling, they were first stuck on glass substrates prior to the above measurements.

## Results and discussion

**Figure 1:**
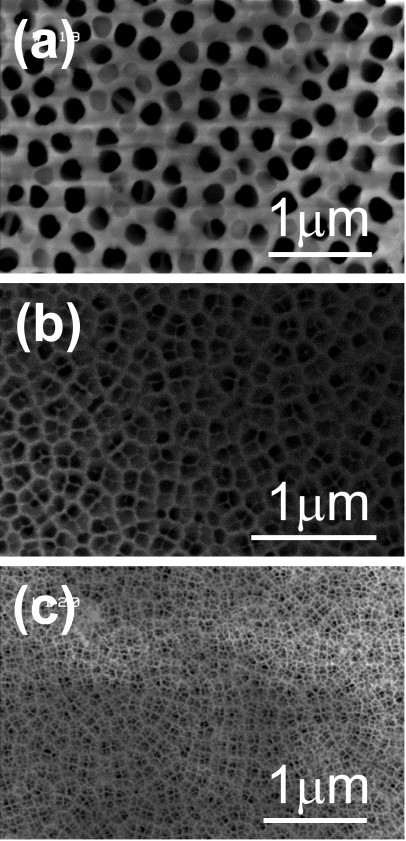
**SEM images of the AAO templates with average pore diameters**. (**a**, **b**, **c**) Average pore diameters around 185, 95, and 40 nm, respectively.

**Figure 2:**
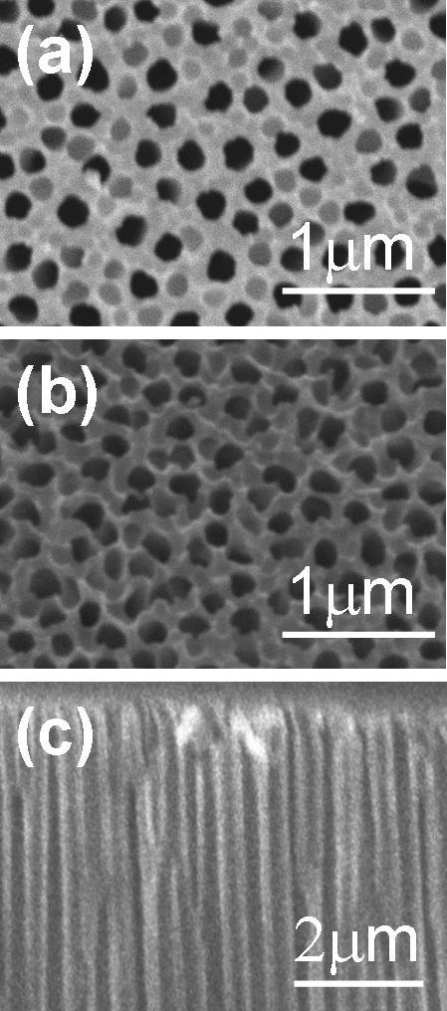
**SEM images of Co/Pd multilayers on AAO templates with average pore diameter of 185 nm**. (**a**, **b**, **c**) The top-view SEM image, bird's-eye view SEM image that was angled at 30°, and cross-sectional SEM image, respectively.

The nanoporous AAO templates were first examined using the SEM. Figure 1 showed the SEM images of the AAO templates with different pore diameters. From the SEM images, we got the average pore diameters of the AAO templates which were around 185, 95, and 40 nm for Figure 1a, b, c, respectively. Figure 2 showed the SEM images of Co/Pd multilayers grown on the AAO template with an average pore diameter of around 185 nm. The top-view SEM image of the Co/Pd sample, Figure 2a, indicated that the Co/Pd film was grown on the interpore region of the AAO template. Irregular Co/Pd grains can be observed in the bird's-eye view SEM image that was angled at 30°. The cross-sectional SEM image of the Co/Pd multilayers was showed in Figure 2c.

**Figure 3:**
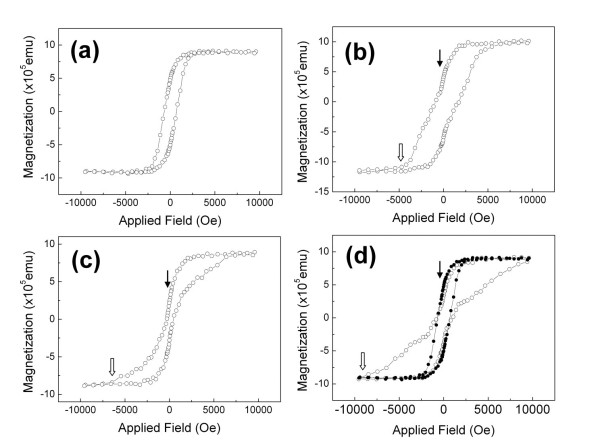
**The perpendicular hysteresis loops of the Co/Pd multilayers**. (**a**) The multilayers grown on the Si substrate. (**b**, **c**, **d**) The multilayers grown on the AAO templates with pore diameters of around 185, 95, and 40 nm, respectively.

The out-of-plane hysteresis loop of the Co/Pd multilayers grown on the Si(100) substrate was showed in Figure 3a. A well-defined perpendicular anisotropy with a coercivity = 695 Oe was obtained. The curves with open circles sketched in Figure 3b, c, d denoted the out-of-plane hysteresis loops of the samples grown on the AAO templates with pore diameters of around 185, 95, and 40 nm, respectively. For the samples grown on the AAO templates, they displayed a two-step magnetization reversal. The solid arrows in Figure 3b, c, d pointed the first magnetization reversal of the Co/Pd multilayers. For clarity, we superimposed the hysteresis loop of Co/Pt multilayers grown on the Si substrate onto the one grown on the AAO template with a 40-nm pore diameter, as shown in Figure 3d. Note that, in Figure 3d, the curve with solid circles denoted the hysteresis loop of Co/Pt multilayers grown on the Si substrate. In the sample grown on the AAO template, as shown in Figure 3d, there was a second magnetization reversal that took place at the field around -4,000 Oe. Such a two-step magnetization reversal was also observed in the samples grown on the AAO templates with 185- and 95-nm pore diameters, as shown in Figure 3b, c, respectively. Unlike the first reversal, the field range of the second magnetization reversal was broad, and the magnetization kept on reversing until the saturation state was reached.

**Figure 4:**
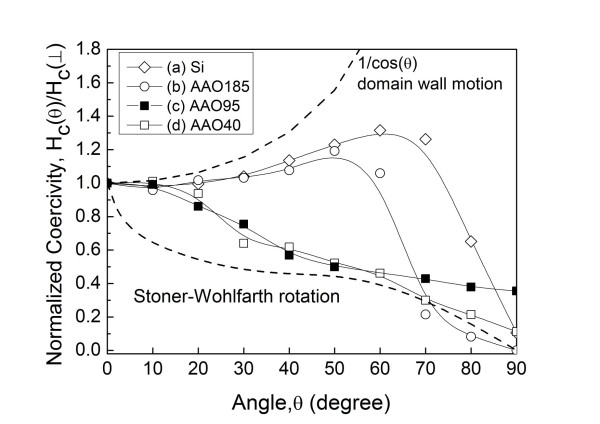
**The normalized coercivity, *H*_c_**(θ)/***H*_c_(⊥), versus the angle, *θ*, of Co/Pd multilayers**. Curve (**a**) is for the multilayers grown on the Si substrate. Curves (**b**, **c**, **d**) are for the multilayers grown on the AAO templates with pore diameters of around 185, 95, and 40 nm, respectively.

The normalized coercivity, *H*_c_(θ)/*H*_c_(⊥), versus the out-of-plane angle, *θ*, as shown in Figure 4 can also give us information about magnetization reversal. Curves (a), (b), (c), and (d) in Figure 4 were for the Co/Pd multilayers grown on the Si(100) substrate and the AAO templates with pore diameters of around 185, 95, and 40 nm, respectively. The dashed curves in Figure 4 were two ideal distributions for the domain-wall mechanism and the rotation only, obeying the Stoner-Wohlfarth [S-W] model. A typical domain wall assisted behavior was observed in the Co/Pd multilayers grown on the Si substrate. For the sample grown on AAO templates with a 185-nm pore diameter, a combination of domain-wall motion and rotation modes was found. In the cases of AAO templates with 95- and 40-nm pore diameters, the S-W rotation dominated the reversal mechanism.

There was no doubt that the first magnetization reversal in Figure 3b, c, d was corresponding to the domain nucleation. The plots of normalized coercivity versus the out-of-plane angle suggested that the second magnetization reversal was a magnetization rotational process. Figure 3b, c, d showed that the magnetization in the second reversal process kept rotating till saturation. As indicated by the open arrows in Figure 3b, c, d, the saturation field of the Co/Pd multilayers increased as the size (or density) of nanopores of the AAO templates was getting smaller (or higher). Therefore, the magnetization rotation that took place in the Co/Pd multilayers can be realized by the pinning effect provided by the underlying AAO nanopores.

**Figure 5:**
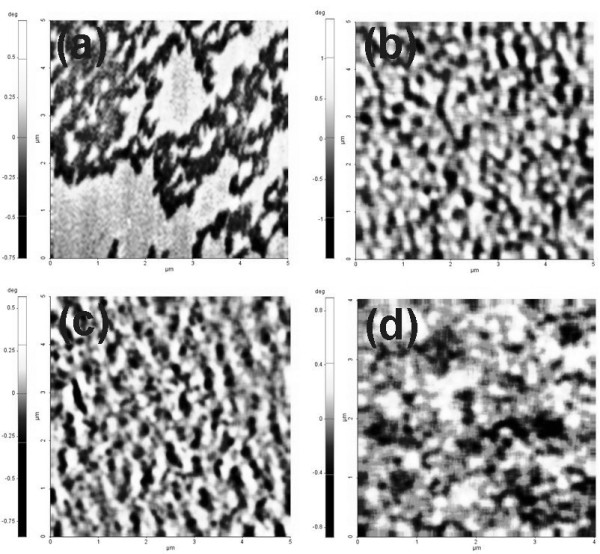
**The magnetic domain images of the Co/Pd multilayers**. (**a**) The multilayers grown on the Si substrate. (**b**, **c**, **d**) The multilayers grown on the AAO templates with pore diameters of around 185, 95, and 40 nm, respectively.

The magnetic domain image of Co/Pd multilayers grown on the Si substrate, shown in Figure 5a, displayed a mazed distribution. It supported a reversal mechanism of domain-wall motion. Short stripe-like domains formed by joining neighboring circular domains were observed in the sample grown on the AAO template with a 185-nm pore diameter, as shown in Figure 5b. Pettier magnetic domains were observed in the samples grown on the AAO templates with pore diameters around 95 and 40 nm, as shown in Figure 5c, d. Manifestly, the domain size of the Co/Pd multilayers decreased as the size (or density) of nanopores of the AAO templates was getting smaller (or higher). We can infer that the domain size was limited by the underlying nanoporous structure. This is also evidence that the nanoporous structure of AAO templates provided a pinning effect.

## Conclusions

We systematically investigated the magnetization reversal of [Co(2 Å)/Pd(10 Å)]_15 _multilayers on the AAO templates with different pore sizes. The magnetization reversal, domain size, and saturation field of the Co/Pd multilayers were strongly influenced by the pore diameter (or density) of underlying AAO templates. The underlying AAO template provided a pinning effect that results in a rotational reversal and a limitation of domain growth. This study demonstrated the magnetization reversal of a percolated recording media with perpendicular anisotropy mainly dominated by the rotational mechanism.

## Competing interests

The authors declare that they have no competing interests.

## Authors' contributions

C-CH fabricated the Co/Pt multilayers. C-CY carried out the MFM images of the samples and drafted the manuscript. S-Y and Y-D participated in the measurements of the hysteresis loops. J-Y carried out the SEM images of the samples. All authors read and approved the final manuscript.
